# Different correlations between lymphocyte subsets from patients with intra-abdominal sepsis and pneumonia-derived sepsis

**DOI:** 10.1186/cc9680

**Published:** 2011-03-11

**Authors:** TS Skirecki, UZ Zielińska-Borkowska, MZ Złotorowicz, JK Kawiak, GH Hoser

**Affiliations:** 1 Medical Center of Postgraduate Education, Warsaw, Poland

## Introduction

Although there has been progress in understanding the immunopathology of sepsis, the mortality rates remain high and there is still a lack of effective immunomodulatory therapies. Possible reasons include heterogeneity of septic patients and inefficiency of methods of monitoring the immune system status [1]. Most of both the experimental and clinical studies do not distinguish sepsis based on the primary sites of infection. Therefore, we studied the differences in the cellular immune response during sepsis originating from pneumonia and peritonitis.

## Methods

Blood samples were obtained from 34 patients treated in our ICU in the first days of sepsis, severe sepsis or septic shock. Intra-abdominal sepsis (IAS) was diagnosed when SIRS symptoms with intra-abdominal, postoperative infection source occurred. Pneumonia-derived sepsis (PDS) diagnosis was based on SIRS accompanied by CXR lung consolidation. Samples were stained with the panel of antibodies against: CD45/CD14, CD3/CD19, CD3/CD4, CD3/CD8, CD3/CD16+56 and isotypic control. Cells were analysed by flow cytometry and total cell count per microliter was calculated. Comparative and simple regression statistical analyses were performed.

## Results

Fourteen patients were diagnosed with IAS and eight with PDS. Etiology of most IAS was Gram-negative, while Gram-positive in PDS. The mortality rate was higher in PDS. Monocyte absolute number and white blood count were the only variables with statistically significant differences between IAS and PDS. The correlations between number of lymphocytes and monocytes, CD3^+^, CD4^+ ^and CD19^+ ^were high in both groups of patients. However, in IAS no correlation was found between the number of either cytotoxic CD8 lymphocytes and NK cells with lymphocyte count. Interestingly, a high correlation for the number of CD8^+ ^and NK cells exists in both IAS and PDS patients.

## Conclusions

Our results indicate differences in the immune response during sepsis originating from respiratory and abdominal infections. Independent correlations between NK cells and cytotoxic lymphocytes suggest existence of shared mechanisms of their regulation.
